# Nanoemulsion curcumin injection showed significant anti-inflammatory activities on carrageenan-induced paw edema in Sprague-Dawley rats^☆^

**DOI:** 10.1016/j.heliyon.2023.e15457

**Published:** 2023-04-14

**Authors:** Adilah Marwa, Iskandarsyah, Mahdi Jufri

**Affiliations:** Laboratory of Pharmaceutics and Pharmaceutical Technology, Faculty of Pharmacy, University of Indonesia, Depok, 16424, Indonesia

**Keywords:** Curcumin, Nanoemulsion, Anti-inflammatory, Intravenous injection, Palm oil (LCT oil)

## Abstract

Medicinal plants are candidates for the discovery of potential new anti-inflammatory agents. Curcumin is the active compound found in turmeric root, which has high anti-inflammatory activity. One of the limitations of curcumin as a therapeutic agent is its low solubility in water and extensive first-pass effect metabolism. The aim of this study was to formulate curcumin nanoemulsion for parenteral injection. We prepared curcumin nanoemulsions with a homogenizer using three surfactant concentrations (1.8%; 2.4%; and 3%) and two curcumin concentrations (1% and 3%). Formulas were evaluated for droplet diameter, polydispersity index, zeta potential, viscosity, pH, entrapment efficiency (EE), osmolality, sterility, and morphology. The nanoemulsion containing 1% curcumin and 3% surfactant (F3) demonstrated good stability. Curcumin nanoemulsions at 20 and 40 mg/kg doses showed anti-inflammatory activity on carrageenan-induced paw edema in male Sprague-Dawley rats. These two doses inhibited paw edema by 33% and 56% respectively at 5 h after carrageenan induction. Inhibition of edema volume by curcumin nanoemulsion at a dose of 40 mg/kg did not show a significant difference (*P* > 0.05) compared to the activity of the standard drug ketorolac at a dose of 2.7 mg/kg. We conclude that curcumin nanoemulsion has anti-inflammatory activity and can be a promising anti-inflammatory agent.

## 1 Introduction

Inflammation is an adaptive response caused by stimulation and tissue damage triggered by infection, damaging chemicals, or physical trauma. The inflammatory response functions to destroy, reduce, and maintain the homeostatic function of the body [1]. Nonsteroidal Anti-inflammatory Drugs (NSAIDs) are drugs that are often used to reduce pain and inflammation. However, the use of NSAIDs triggers various side effects such as gastrointestinal ulcers, stomach ulcers, excessive bleeding, and perforation at the level of the digestive tract [2]. Thus, the development of new anti-inflammatory drugs is still needed to overcome the side effects of NSAIDs. Research on the pharmacological activity of medicinal plants is important and continues to be carried out because medicinal plants contain biologically active compounds with fewer side effects [3].

Curcumin is a polyphenolic compound contained in turmeric (*Curcuma longa,* Family: Zingiberaceae). Curcumin is widely used as natural medicine because it has various physiological activities such as an anti-inflammatory [4], antioxidant, and anticancer drug [5]. Several studies have reported that curcumin has a high potential to be used as an anti-inflammatory drug. One drawback of curcumin as a therapeutic agent is its low water solubility which causes low curcumin solubility and adsorption in the bloodstream. In addition, curcumin is extensively destroyed in first-pass metabolism. The results of first-pass metabolism produce metabolites from curcumin derivatives in the form of dihydrocurcumin and tetrahydrocurcumin, which have the same efficacy as curcumin but with a much lower therapeutic effect [6]. Alternative routes of administration and development of curcumin formulations are needed to overcome these problems.

The parenteral route is the most effective route of drug delivery for active substances that extensively pass first-pass metabolism. Administration of curcumin via the intravenous route can significantly increase the absorption of curcumin compared to oral administration [7]. However, active substances such as curcumin with low solubility are relatively unsuitable for parenteral administration. Emulsification is a method used to modify the solubility properties of lipophilic drugs [8].

One of the emulsion preparations that has high stability is nanoemulsion. It is because the nanoemulsion has a small size so that it does not experience general emulsion problems such as creaming, coalescence, sedimentation, and flocculation. In addition, a particle size of less than 500 nm can reduce the risk of pain on injection and better target localization. Several factors that affect the stability and bioaccessibility of curcumin nanoemulsion carriers are the nature of the emulsifier, the type, and the amount of carrier oil [9].

Indonesia is one of the largest palm oil producer countries in the world. As a vegetable oil, palm oil is a promising source of industrial fats and oils because it has many advantages, such as high thermal stability, high productivity, and good oxidative stability compared to other parenteral emulsion oils [10]. The study used a combination of carrier oil phases. The combination of palm oil (LCT) and coconut oil (MCT) can increase fatty acid metabolism, reduce the toxicity of LCT in palm oil, and is more stable to extreme temperatures [10,11].

There have been many publications regarding curcumin-based drug delivery systems such as oral and transdermal curcumin nanoemulsion formulations as wound healing and anti-inflammatory, formulation and characterization of curcumin encapsulating nanoemulsions, curcumin loaded PEGylated nanoemulsions as antioxidants, development of food-grade curcumin nanoemulsions as antioxidants [[12], [13], [14], [15]]. However, most studies focused on assessing the potential effects of curcumin, developing non-parenteral nanoemulsion formulas, and only a few publications have focused on developing parenteral delivery route curcumin nanoemulsions for anti-inflammatory. This study developed a new formula of curcumin nanoemulsion with a combination of palm oil and MCT oil for the intravenous injection route. One of the main requirements for parenteral preparations is sterile, but the sterilization process can cause instability of the nanoemulsion [16]. The nanoemulsion stability and the pharmacodynamic effects of anti-inflammatory curcumin nanoemulsion injection were assessed in this study.

## 2 Materials and methods

### 2.1 Materials

All reagents used in this study were obtained from commercial suppliers and used without further purification. The curcumin used to make the preparations was curcumin 97% (Cur) purchased from Xi'an Natural Field Bio-Tech China, and Standard curcumin (99%) purchased from Sigma Aldrich. The materials for the vehicle are palm oil (Asian Chemical), MCT coconut oil (PT. Okusi Biotech), egg lecithin (GMBH, Germany), sodium oleate (GMBH, Germany), glycerin (Brataco), and other materials such as ethanol 96%, N_2_ gas, ketorolac tromethamine, NaCl 0.9%, double distilled water (Ikapharmindo), methanol (HPLC grade), acetonitrile (HPLC grade), acetic acid glacial (HPLC grade), and 1 ml injection syringe purchased from a local pharmaceutical store.

### 2.2 Animals

Sprague-Dawley male rats (200–250 g) obtained from the Central Laboratory of the Faculty of Medicine (Padjadjaran University, Bandung) were used in this study. Rats were acclimatized at the animal facility of the Faculty of Pharmacy, University of Indonesia, under standard temperature conditions (25±2 °C), fed, and watered ad libitum for two weeks. At the end of each experiment, animals were sacrificed to a lethal dose of ketamine. The research protocol was approved by the Health Research Ethics Committee, Faculty of Medicine, University of Indonesia.

## 3 Method

### 3.1 Preparation of nanoemulsion curcumin

The nanoemulsions were prepared by formulating the oil and water phases separately. Curcumin, a combination of oil and surfactants were prepared into six formulas with the ratios shown in Table 1. The aqueous phase was created by mixing glycerin (2.5%), sodium oleate (0.03%) and aquabidest in a beaker glass and then stirred with a magnetic stirrer while heated to a temperature of 70 °C. The oil phase was prepared in different beakers by mixing lecithin, palm oil, and MCT Coconut Oil and then stirred with a magnetic stirrer while heated to a temperature of 70 °C. Furthermore, the water phase, oil phase, and active substance were homogenized using a homogenizer at a speed of 10,000 rpm. Then the coarse emulsion was reduced using the ultrasonication method with a frequency of 60 Hz for 5 min, forming a nanoemulsion. Curcumin has poor stability at alkaline pH, so the nanoemulsion preparation is maintained at pH 6.2 by adding citrate buffer. In addition, curcumin, palm oil, and lecithin are easily oxidized. Therefore, N_2_ gas was purged into the mixture for ±5 min to prevent oxidation. Finally, the nanoemulsions were sterilized using an autoclave at a temperature of 121 °C for 15 min [16].

**Table 1:** Composition, size, polydispersity index, zeta potential, phase separation, viscosity, pH values nanoemulsion.

Formula	Cur (%) (v)	Lct (%) (v)	Oil (%)	Particle Size (nm)	Polidispersity Index	Zeta Potential (mV)	Phase separation	Viscosity	pH
F1	1	1.2	10	359.67 ± 20.42	0.20 ± 0.02	−28.43 ± 1.94	Not observed	5.97 ± 0.17	6.02 ± 0.03
F2	1	2.4	10	333.67 ± 8.26	0.23 ± 0.11	−35.27 ± 0.56	Not observed	6.43 ± 0.21	6.04 ± 0.04
F3	1	3	10	322.67 ± 11.26	0.13 ± 0.01	−41.2 ± 0.98	Not observed	6.63 ± 0.25	6.05 ± 0.05
F4	3	1.2	10	233.33 ± 27.13	1 ± 0.00	−28.03 ± 2.29	Observed	15.53 ± 0.21	6.09 ± 0.04
F5	3	2.4	10	218.33 ± 113.11	0.88 ± 0.10	−27.33 ± 1.86	Observed	16.27 ± 0.23	6.10 ± 0.07
F6	3	3	10	138.67 ± 37.81	0.55 ± 0.13	−23.57 ± 2.24	Observed	18.89 ± 0.35	6.10 ± 0.03

*Cur, curcumin; Oil, palm oil and MCT oil; Lct, egg lecithin. Data are mean values (n = 3) ±SD.

### 3.2 Characterization of curcumin nanoemulsions

#### 3.2.1 Particle size analysis

The particle size distribution and the average droplet diameter (nm) of the nanoemulsion were observed using a Malvern zeta sizer (Nano ZS, Malvern instrument, UK) which operates on the principle of dynamic light scattering. Measurements were conducted on the six formulas to observe particle size, PDI, and zeta potential. The samples were diluted and placed in disposable zeta cells. A sample solution with a minimum volume of 1 ml was added dropwise into a size cell or a cuvette until the concentration was sufficient, after which the nanoemulsion particles were measured [17]. Light scattering was monitored at an angle of 90°, at 25 °C. The analysis was repeated in triplicate and the significance of the differences was analyzed.

#### 3.2.2 Centrifugation test

Centrifugal stability was tested by a high speed refrigerated centrifuge (Suprema 21, Japan). 10 ml of the nanoemulsion preparation was placed into a centrifugation tube and then centrifuged at 15,000 rpm at 4 °C for 60 min. The presence or absence of phase separation was then observed [18].

### 3.3 Viscosity, rheology properties, and pH

The viscosity test was carried out using Brookfield Rheometer at 25 ± 0.5 °C, with a spindle RV 1. The pH of the nanoemulsions was measured using a calibrated pH meter (550 pH meter, Jenway, UK) at 25 °C ± 1 °C. The measurements were carried out in triplicates.

#### 3.3.1 Entrapment efficiency

The entrapment efficiency test was carried out on formulas with good stability test results. The nanoemulsion was placed into a 2 ml centrifuge tube and then centrifuged at 15,000 rpm at 5 °C for 60 min. After that, 5 μl of supernatant was taken. The supernatant was then diluted by adding 5 ml of methanol. 10 μl of this solution was injected into the HPLC Shimadzu LC 20AD. The sample was analyzed in a reversed-phase HPLC using acetonitrile and glacial acetic acid 2% (50:50 v/v) as mobile phase and C18 column stationary phase with a flow rate of 1.2 ml/min. The analysis was detected using UV detector at 425 nm wavelength and a column temperature of 25 °C. The percentage of drug that is not entrapped can be calculated using the following Eq. (1):

(1) $$\%EE=\left(\frac{Total\;amount\;of\;Cur-Total\;amount\;of\;Cur\;entrapped}{Total\;amount\;of\;Cur}\right)\times 100$$

#### 3.3.2 Osmolality measurement

The osmolality of the curcumin nanoemulsion was measured using an osmometer based on the principle of the freezing point method. The osmometer was calibrated with reference standards of 100 and 3000 mOsm/kg (Advance Instruments, Inc.). A total of 0.25 ml of sample was taken using a micropipette and then analyzed. Measurements were repeated three times [17].

#### 3.3.3 Sterility study

Plate Count Agar (PCA) media and Potato Dextrose Agar (PDA) media were used in sterility test, respectively. 0.1 ml of nanoemulsion was added to each medium and made in duplicate. The petri dishes for PCA media were incubated at 35–37 °C and petri dishes for PDA media were incubated at 28 °C. Both were incubated for 24–48 h in an inverted position and observed for the presence or absence of fungal colony growth. The sterility test was carried out by calculating the total plate number and the number of yeast molds growing in the curcumin nanoemulsion preparation [19].

##### 3.3.3.1 Transmission electron microscopy

Nanoemulsion morphology was tested using a transmission electron microscope (TEM; JEM 1400, JEOL, Tokyo, Japan). A total of 5 μl of the sample was dropped into the specimen holder and covered with a 400 mesh grid. After 1 min, 0.5% uranyl acetate solution was dripped onto the grid, and this sample was allowed to dry for 30 min before being observed under an electron microscope at magnification [20].

#### 3.3.4 Accelerated stability test

Accelerated stability studies were carried out on an optimized curcumin nanoemulsion (F3) according to International Conference on Harmonization (ICH) guidelines. The physical stability test of the nanoemulsion was carried out by observing the changes in appearance, content, pH, sterility, phase separation, viscosity, particle size, polydispersity index, and zeta potential. Nanoemulsions were stored at room temperature (25 ± 2 °C) for 12 weeks and observed at weeks 0, 4, 8, and 12 [21].

#### 3.3.5 Anti-inflammatory effect test of curcumin nanoemulsion

##### 3.3.5.1 Anti-inflammatory test procedure

This study used the Winter method [22] that was modified by subplantar injection of 1% carrageenan solution. Twenty male Sprague-Dawley rats (200–250 g) were randomly divided into 4 groups (n = 20). All rats were not fed for 24 h before the experiment but were still allowed to drink. 0.2 ml of 1% carrageenan solution was injected to the negative control group at the subplantar site. The positive control group was given an intravenous injection of ketorolac (2.7 mg/kg), and groups I and II were given an intravenous injection of curcumin nanoemulsion with a dose of 20 mg/kg and 40 mg/kg respectively. Five minutes after the intravenous injection, the positive control group and the experimental group were injected with 0.2 ml of 1% carrageenan solution via subplantar. Paw edema volume was measured at 1, 2, 3, 4, 5, and 6 h after carrageenan injection using a plethysmometer. The average edema inhibition in the test group was calculated by Eq. (2):

(2) $$\%Inhibition=\left\{1-\frac{\left(a-x\right)}{\left(b-y\right)}\right\}\times 100\%$$

Eq (2). Edema inhibition formula; a = paw volume before carrageenan injection on the test group; b = paw volume each hour after carrageenan injection on the test group; x = paw volume before carrageenan injection on the negative control group; y = paw volume each hour after carrageenan injection on the negative control group.

### 3.4 Statistical analysis

All data from paw edema volumes were expressed as means (n = 5). Values of edema inhibition were expressed as a percentage and compared with the control group using one way-ANOVA (*P* < 0.05) followed by a Least Significant Difference (LSD) test. The Kruskal-Wallis test was carried out if one of the requirements for the ANOVA test was not met, and the analysis is continued with the Mann-Whitney test if there was a significant difference. T-test was used to compare variables between the two groups.

## 4 Results and discussion

### 4.1 Particle characterization

In this study, optimization of the nanoemulsion formula was carried out by comparing the concentrations of curcumin and surfactants as shown in Table 1. The produced nanoemulsion from all formulas was characterized and compared in terms of average particle size, polydispersity index (PDI), and zeta potential. The nanoemulsion from the six formulas showed a globule size of <500 nm [23]. The small globule size causes the Brownian motion to occur faster and, thus, prevents the sedimentation process [24]. The results showed that increased surfactant concentration would result in a smaller particle size. Particle size <500 nm is considered to be able to reduce pain on the injection [25].

The value of the polydispersity index (PDI) indicates the level of uniformity of a sample. A PDI value of more than 0.5 indicates a heterogeneously distributed sample [26]. Formula 4, 5, and 6 showed a PDI value higher than 0.5, which means that the globules tend to form aggregates. Formula 1, 2, and 3 showed PDI values of less than 0.5 which indicated that the globules from the three nanoemulsion formulas showed a narrow size distribution. Zeta potential measurements were carried out on the six nanoemulsion preparations in order to determine the nature of the electric charge of the nanoparticles.

**Fig. 1:**
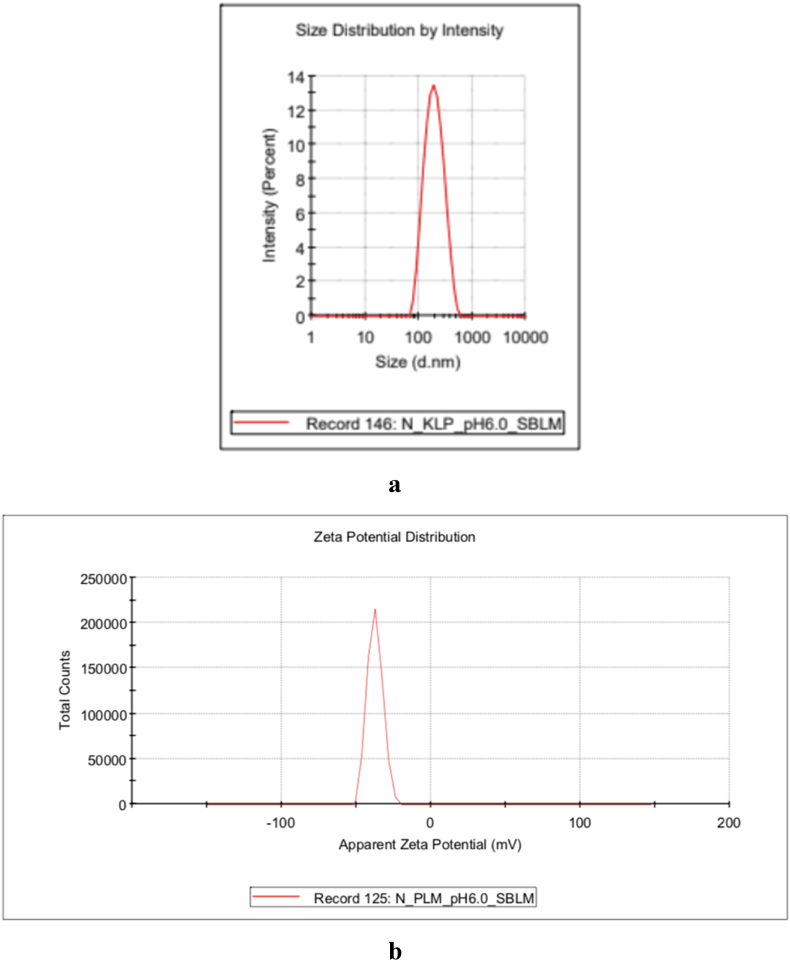
Particle size and distribution (nm) (a) and zeta potential (mV) (b) measured from nanoemulsion Formula 3

The zeta potential gives an illustration of the repulsive force between particles. The greater value of the zeta potential will give a more stable dispersion system [25]. A dispersion system with a low zeta potential value will easily form aggregates along with Van der Waals forces in the particle interactions [27]. The zeta potential value of nanoparticles less than −30 mV or greater than +30 mV is considered to have higher stability [28]. Based on the measurement results, the six formulas demonstrated a negative zeta potential [29]. It was also found that only formulas 2 and 3 showed zeta potential values of less than −30 mV. These results indicated that the three nanoemulsion formulas had fairly good stability and tended not to form aggregates easily. Formula 3 showed the smallest particle size, PDI, and zeta potential (Fig. 1(a and b)).

#### 4.1.1 Physical stability test and entrapment efficiency

**Fig. 2:**
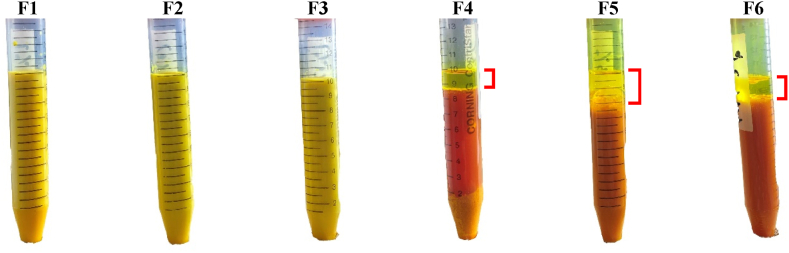
The separation phase nanoemulsion curcumin to determine the physical stability after the centrifugation test.

The principle of the physical stability test by centrifugation is that the force of gravity can accelerate the sedimentation rate and, thus, cause phase separation of the nanoemulsion. The centrifugation test described the stability of the nanoemulsion during one-year storage [30]. Based on Fig. 2, separation occurs in formulas 4, 5, and 6, probably due to the high concentration of curcumin and the concentration of the surfactant used does not have a strong ability to bind the oil phase and the water phase. The entrapment efficiency test results for Formula 4, 5, and 6 were 33.14 ± 3.52%, 33.87 ± 2.49%, and 42.79 ± 1.10%.

Formula 1, 2, and 3 showed good stability during one year of storage. The entrapment efficiency test results for Formula 1, 2, and 3 were 72.10 ± 5.03%, 88.29 ± 0.34%, and 96.57 ± 1.0%. These results indicate that the higher the surfactant concentration, the higher the percentage of drug adsorbed. Optimal absorption occurs in Formula 3.

#### 4.1.2 Viscosity, rheology, and pH measurement

Viscosity is the measure of a fluid's resistance to flow, where highly viscous fluid has a high resistance to flow [31]. Viscosity measurement was carried out only at room temperature in order to minimize temperature effects [11]. Viscosity measurements carried out at high temperatures will cause the viscosity of the nanoemulsion to be lower than it should be. In contrast, if it is measured at low temperatures, it will cause the viscosity to be higher than it should be. The viscosity measurement results are depicted in Table 1. Increasing the concentration of curcumin (F4, F5, and F6) led to increased viscosity of the nanoemulsion. The increase in viscosity is probably due to uneven distribution size of the nanoemulsion globules caused by the poor solubility of curcumin. The viscosity of F1, F2, and F3 are not significantly different. The difference in the viscosity value of each preparation is probably affected by the amount of surfactant concentration in the formula. Incorporation of more surfactant to the nanoemulsion system can increase the viscosity [32]. The use of coconut oil as part of the oil phase and also the low percentage of the oil phase is the main contributor to the low viscosity value of the emulsion [33]. The produced curcumin nanoemulsion also showed a non-Newtonian flow type, dilatant, which showed increasing shearing stress as the rate of shear elevated. The particles in the liquid with this type of flow will move quickly among each other to occupy the empty space.

The measurement of nanoemulsion pH was carried out to determine the stability level of curcumin nanoemulsion preparations and the safety of using curcumin nanoemulsion injections. The pH of the preparation was maintained at 6.0 because curcumin degraded at an alkaline pH [34]. The six nanoemulsion formulations showed pH value of 6–8, considered to be the optimum pH range for intravenous emulsions [35].

#### 4.1.3 Nanoemulsion morphology

**Fig. 3:**
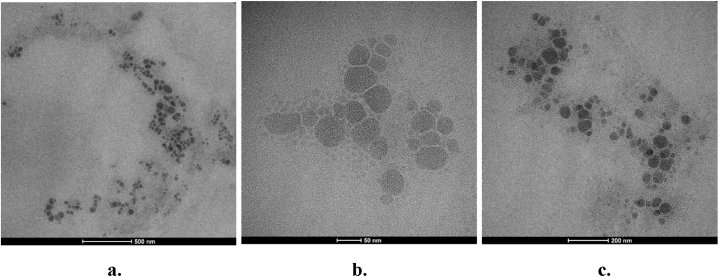
Particle size distribution of formula 3 by TEM. (a) 15.000×, (b) 71.000×, (c) 97.000×

Observation of globule morphology in this study was carried out on nanoemulsion formula 3. The morphology of the nanoemulsion can be seen microscopically using TEM, as shown in Fig. 2. Images obtained from TEM can be used to support the result of particle size measurement using a particle size analyzer. The TEM images shown in Fig. 3(a–c) demonstrated the spherical shape of nanoemulsion with the active substance in the inner part of the globules. The spherical globule is considered able to penetrate cellular membranes easily and can reduce aggregation because the interaction between the particle surfaces is reduced [36]. TEM imaging with magnification 97.000 times showed that nanoemulsion has a globule size of about 300 nm (Fig. 3c). This result is consistent with the particle size value in Formula 3. Nanoemulsions have a high free energy difference between the separated phases so they tend to contain spherical particles due to the high Laplace pressure working on them [37]. The nanoemulsion's spherical ball-like appearance indicates that the particles are evenly dispersed.

#### 4.1.4 Osmolality test

According to the literature, the osmolality of drug products should ideally be controlled at a level of around 300 ± 30 mOsm/kg [17]. However, for drug products intended for intravenous or intravascular injection, the recommended upper osmolality limit is generally below 1000 mOsm/kg for small-volume injections (100 ml). If the osmolality value is less than 150 mOsm/kg, it can cause hemolysis. In this study, the osmolality of nanoemulsion formula (F3) was found to be 545,6 ±15,9 mOsm/kg. This value is still within the acceptable hypertonic range for intravenous injection [38].

#### 4.1.5 Sterility test

**Fig. 4:**
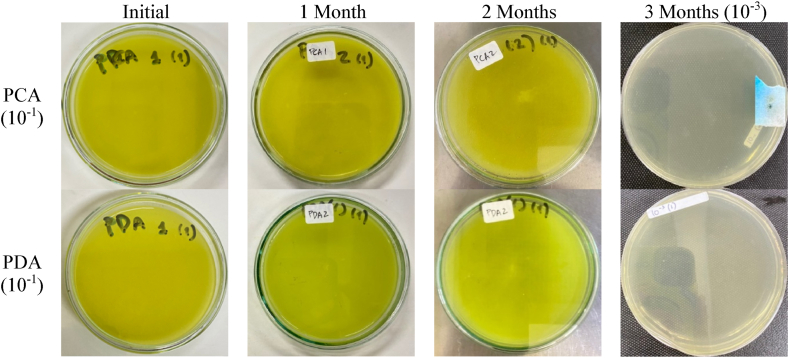
Sterility test of curcumin nanoemulsion on PCA and PDA media after 5 days of incubation.

The production of curcumin nanoemulsion in this study used final sterilization (autoclave) and was carried out aseptically. This was due to at the beginning of the experiment, and we did not obtain sterile curcumin nanoemulsions because we only carried out the final sterilization. Thus, we carried out several formula optimizations to make a sterile and stable curcumin nanoemulsion after the final sterilization process. The sterility test was carried out on the most stable formula (F3) using PCA and PDA media shown in Fig. 4 The results showed no growth of bacteria on the media after five days of incubation.

Based on the data in Appendix 4. The statistical test using *t-test* showed no significant effect of temperature in the sterilization process to particle size or polidispesity index (*P* > 0.05), but there was a decrease in pH. Nanoemulsions containing phospholipids tend to be more stable to heat, so there is no increase significantly in particle size or polydispersity index [39]. Meanwhile, the decrease in pH can be caused by releasing free fatty acids in the emulsion from the oil phase or emulsifier [40].

#### 4.1.6 Accelerated stability test

Accelerated stability tests show how the drug quality varies over time, affected by various factors, such as temperature, humidity, and light. Drug products should be fully characterized physically, chemically, and microbiologically at the start of research and throughout the desired shelf life [21]. The results of the research in Table 2 show that there was no significant change in all test parameters after 90 days. There was an increase in zeta potential, particle size, polydispersity index, viscosity, and a decrease in the pH of the nanoemulsion starting from month 1 to month 3. The decline in pH and increase in zeta potential was attributed to the hydrolysis of the egg lecithin and oil from the emulsion due to temperature. Increasing temperature change the phosphatidyl choline and phosphatidyl ethanol amine to form lyso to lower the emulsion's pH [41,42]. An increase in temperature will increase the kinetic energy of the dispersed phase, causing an increase in globule diameter [43]. An increase in globule diameter causes an increase in particle size, polydispersity index, and emulsion viscosity.

**Table 2:** Size, polydispersity index, zeta potential, sterility, viscosity, pH values nanoemulsion, sterility.

Formula 3	Particle Size (nm)	Polidispersity Index	Zeta Potential (mV)	Sterility	Viscosity	pH	Curcumin content
0 Month	322.67±11.26	0.13 ± 0.01	−41.2 ± 0.98	Sterile	6.63 ± 0.25	6.05 ± 0.05	99.63% ± 2.65
1 Month	347.67± 20.03	0.29 ± 0.05	−38.7 ± 0.25	Sterile	6.88 ± 0.31	6.00 ± 0.03	99.14% ± 0.62
2 Month	348.67± 14.67	0.33 ± 0.002	−38 ± 0.10	Sterile	7.56 ± 0.27	5.89 ± 0.04	97.52% ± 1.61
3 Month	388.33± 16.50	0.36 ± 0.024	−36.87 ± 0.32	Sterile	8.43 ± 0.25	5.50 ± 0.05	96.52% ± 2.59

*Data are mean values (n = 3) ±SD.

Nonetheless, the statistical test using Mann-Whitney *U* test showed no significant changes (*P* > 0.05) in particle size, polydispersity index, viscosity, and curcumin content observed over three months. The excellent stability can be due to the steric stabilizing effect of the non-ionic emulsifier (lecithin) whereby a bulk steric barrier is formed against the colliding particles. Thus, this phenomenon prevents flocculation and coalescence. In addition, it has been reported that MCT can destabilize emulsions due to droplet aggregation, whereas LCT can increase MCT viscosity and emulsion particle size distribution, which will improve emulsion stability during storage [44]. Based on the sterility test on nanoemulsion formula (F3), both media had no microbial growth after incubation. The results showed that the resulting nanoemulsion formula met the criteria for a sterile preparation for three months of shelf life.

#### 4.1.7 *In vivo* anti-inflammation study

The negative control group rats experienced an increase in edema volume which could be observed within 1 h and reached its peak 5 h after induction [45]. The first phase of inflammation caused by carrageenan induction began 1.5 h after induction. At this hour, histamine and serotonin are produced, then followed by the release of bradykinin 1.5 h–2.5 h later. The last phase, commonly called the second phase, occurs between 2.5 and 6 h. In the second phase, inflammatory mediators of prostaglandins which were mediated by polymorphs (neutrophils and monocytes) were released. Based on this, an analysis of the entire group was carried out at 2 h, 3 h, 4 h, and 5 h.

**Fig. 5:**
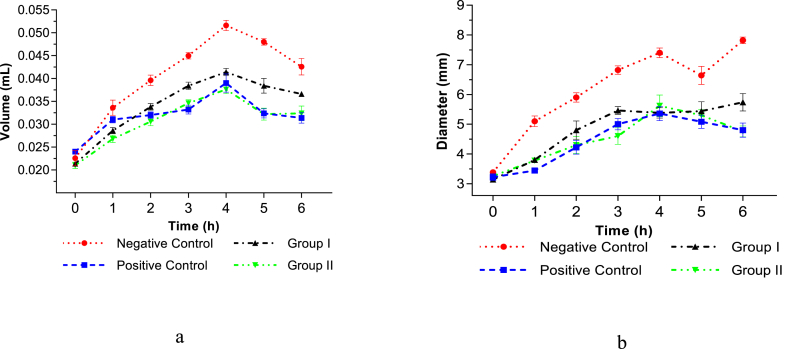
(a) Rat paw edema volume of the negative control group, positive control group, group I, and group II at 0–6 h. (b) Rat paw edema diameter of the negative control group, positive control group, group I, and group II at 0–6 h. Values are expressed as mean values ± (n = 5).

For the in vivo study, F3 was chosen since it demonstrated good nanoemulsion stability. The doses used in the study group were 20 mg/kg BW and 40 mg/kg BW, and the positive control group was given an injection of 2.7 mg/kg BW of ketorolac. The drug was given intravenously 5 min prior to carrageenan induction. In Fig. 5(a and b) it can be seen that the administration of 2.7 mg/kg BW of ketorolac, 20 mg/kg BW of curcumin and 40 mg/kg BW can significantly reduce the volume and diameter of rat paw edema *(P* < 0.05). Since the result is similar to ketorolac, a well-known anti-inflammatory drug, the reduced volume of this paw indicates that the 20 and 40 mg/kg BW curcumin nanoemulsions are proven to have anti-inflammatory activity. Statistical analysis using One-way ANOVA followed by Tukey's post hoc analysis did not show a significant difference (*P* > 0.05) of curcumin nanoemulsion injection at 40 mg/kg BW compared to the positive control at 2, 3, 4, and 5 h. This indicated that curcumin nanoemulsion at a dose of 40 mg/kg BW had an anti-inflammatory effect similar to ketorolac.

**Fig. 6:**
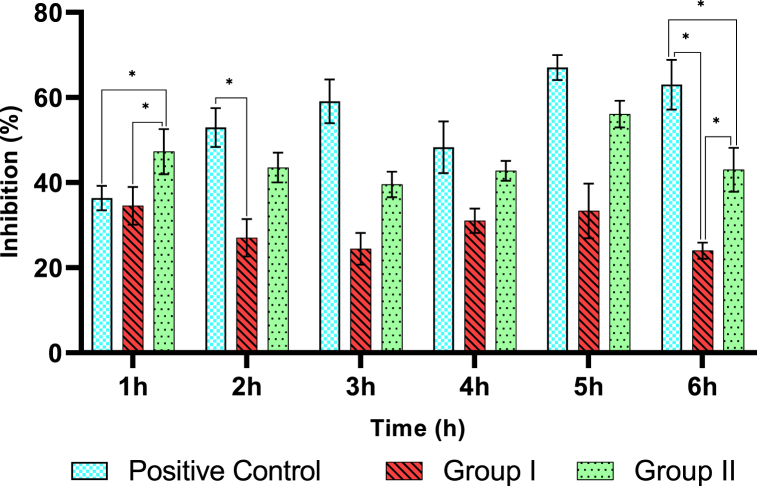
Percent (**%)** Inhibition of rat paw edema volume of the negative control group, group I, and group II at 1–6 h. Values are expressed as mean values ± (n = 5). Values followed by an asterisk (*) are statistically significant difference by Tukey's test (P < 0.05).

**Fig. 7:**
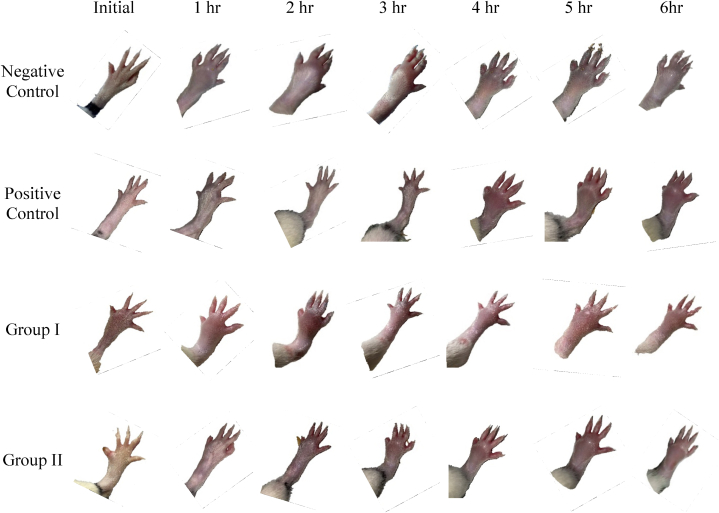
Morphological characteristics of the left hind paw from the various rat groups at 0–6 h.

Fig. 6 showed that the greatest inhibition of inflammation occurred in the positive control, group I, and group II, respectively, 67.06% (5th hour), 33.33% (5th hour), and 56.08% (5th hour). This indicated that the effectiveness of the anti-inflammatory effect of curcumin is affected by the dose, and the injection of curcumin nanoemulsion at a dose of 40 mg/kg BW is more effective as an anti-inflammatory. The anti-inflammatory effect of curcumin nanoemulsion on the rat's paw induced by carrageenan can be seen in Fig. 7.

The ability of curcumin to inhibit swelling in rat feet is due to its ability as an anti-inflammatory. Curcumin has the ability to downregulate various pro-inflammatory mediators, which include prostaglandins, cyclooxygenase 2, lipoxygenase, Nitric Oxide Synthase (iNOS), necrosis factor (TNF-α), interleukins (IL-1, IL-2, IL-6, IL- 8, IL-12) and chemokines. In addition, curcumin showed its ability to block the NF-κB activation pathway, which strengthens its activity as an anti-inflammatory from natural ingredients, and thus, can improve the pathogenesis of inflammation [46].

The curcumin nanoemulsion injection formula has successfully inhibited inflammation in a rat model. This study used a plethysmometer to determine the pharmacodynamic effects on rat paws. To ensure the pharmacodynamic effect and anti-inflammatory mechanism of curcumin nanoemulsion more precisely, it can be determined by measuring the inflammatory mediators present. In addition, FDA safety data for using intravenous injection of curcumin has potential adverse effects such as nausea and vomiting, numbness and tingling in the mouth, skin rash, pain, and swelling at the injection site [47]. The use of injectable curcumin nanoemulsion carriers can reduce pain at the injection site, irritation, and thrombophlebitis [8]. One of the critical points of emulsion carriers for injection is particle size. Particle size could affect the physical stability of the emulsion and the pain during an injection is caused by these factors, so a histological examination of the area induced inflammation and the injection site is necessary. Measurement of inflammatory mediators and histopathological examination can be useful information, so our research team will pursue this goal in further studies.

## 5 Conclusion

The selected curcumin nanoemulsion formula was formula 3 with spherical morphology, the highest percentage of drug adsorption, particle size, polydispersity index, and zeta potential. Stability test results show that the use of a combination of palm oil and MCT oil can produce a stable nanoemulsion. Intravenous injection of curcumin nanoemulsion showed anti-inflammatory activity in carrageenan-induced paw edema. At certain doses, (40 mg/kg BB) curcumin has similar anti-inflammatory activity to standard drugs by the same route. The results of this study indicate that the curcumin nanoemulsion intravenous injection is a potential candidate to be a new anti-inflammatory agent.

## Author contribution statement

Adilah Marwa: Conceived and designed the experiments; Performed the experiments; Analyzed and interpreted data; Wrote the paper. Iskandarsyah: Analyzed and interpreted data; Wrote the paper. Mahdi Jufri: Conceived and designed the experiments; Performed the experiments; Analyzed and interpreted data; Contributed reagent and materials; Wrote the paper.

## Data availability statement

Data included in article/supp. material/referenced in article.

## Additional information

Supplementary content related to this article has been published online at [URL].

## Funding statement

This research was financially supported by Ministry of Education, Culture, Research, and Technology.

## Declaration of competing interest

The authors declare the following financial interests/personal relationships which may be considered as potential competing interests:
