# Supplementary material for: Nanoemulsion curcumin injection showed significant anti-inflammatory activities on carrageenan-induced paw edema in Sprague-Dawley rats
Source: Heliyon. 2023 Apr 14;9(4):e15457. doi: 10.1016/j.heliyon.2023.e15457 (PMC10161698; doi:10.1016/j.heliyon.2023.e15457)
Supplement: Multimedia component 1 [file mmc1.docx]

**APPENDIX**

**Appendix 1. Curcumin Content and Entrapment Efficiency**

1. **Calibration Curve**

| Sample | Area | Average | STD |
| --- | --- | --- | --- |
| 2 ppm | 219401 | 217.401 | 2747.9 |
|  | 214268 |  |  |
|  | 218535 |  |  |
| 4 ppm | 391404 | 394.288 | 4119.7 |
|  | 399006 |  |  |
|  | 392453 |  |  |
| 6 ppm | 606853 | 595.235 | 10131.5 |
|  | 590615 |  |  |
|  | 588237 |  |  |
| 8 ppm | 772023 | 770.701 | 10246.7 |
|  | 780222 |  |  |
|  | 759857 |  |  |
| 10 ppm | 949366 | 949.436 | 1029.3 |
|  | 950499 |  |  |
|  | 948444 |  |  |

y = 92021x + 33287
R² = 0.9995

1. **Curcumin Content**

| Sample | Area | PPM Calculation | PPM Design | Curcumin assay | Mean %Curcumin | Std |
| --- | --- | --- | --- | --- | --- | --- |
| 0 Month | 398.888 | 3.97 | 4 | 99.33% | 99.63% | 2.65% |
|  | 410.276 | 4.10 | 4 | 102.42% |  |  |
|  | 390.876 | 3.89 | 4 | 97.15% |  |  |
| 1 Months | 397.769 | 3.96 | 4 | 99.02% | 99.14% | 0.62% |
|  | 396.198 | 3.94 | 4 | 98.59% |  |  |
|  | 400.666 | 3.99 | 4 | 99.81% |  |  |
| 2 Months | 389.800 | 3.87 | 4 | 96.86% | 97.52% | 1.61% |
|  | 387.888 | 3.85 | 4 | 96.34% |  |  |
|  | 398.999 | 3.97 | 4 | 99.36% |  |  |
| 3 Months | 383.225 | 3.80 | 4 | 95.07% | 96.52% | 2.59% |
|  | 399.576 | 3.98 | 4 | 99.51% |  |  |
|  | 382.856 | 3.80 | 4 | 94.97% |  |  |

1. **Entrapment Efficiency**

| Sample | Area | Calculation PPM | Designed PPM | %EE | Mean | STD |
| --- | --- | --- | --- | --- | --- | --- |
| F1 | 137.760 | 1.14 | 4 | 71.62% | 72.11% | 5.03% |
|  | 116.623 | 0.91 | 4 | 77.36% |  |  |
|  | 153.507 | 1.31 | 4 | 67.34% |  |  |
| F2 | 77.497 | 0.48 | 4 | 87.99% | 88.29% | 0.38% |
|  | 74.795 | 0.45 | 4 | 88.72% |  |  |
|  | 76.857 | 0.47 | 4 | 88.16% |  |  |
| F3 | 47.442 | 0.15 | 4 | 96.15% | 96.57% | 1.00% |
|  | 48.592 | 0.17 | 4 | 95.84% |  |  |
|  | 41.698 | 0.09 | 4 | 97.71% |  |  |
| F4 | 276,509 | 2.64 | 4 | 33.92% | 33.14% | 3.52% |
|  | 268,150 | 2.55 | 4 | 36.19% |  |  |
|  | 293,538 | 2.83 | 4 | 29.30% |  |  |
| F5 | 266,138 | 2.53 | 4 | 36.74% | 33.87% | 2.49% |
|  | 281,632 | 2.70 | 4 | 32.53% |  |  |
|  | 282,312 | 2.71 | 4 | 32.35% |  |  |
| F6 | 239,723 | 2.24 | 4 | 43.92% | 42.78% | 1.10% |
|  | 247,812 | 2.33 | 4 | 41.72% |  |  |
|  | 244,128 | 2.29 | 4 | 42.72% |  |  |

**Appendix 2. Particle Size and PDI Data**

**F1 no.1**

**
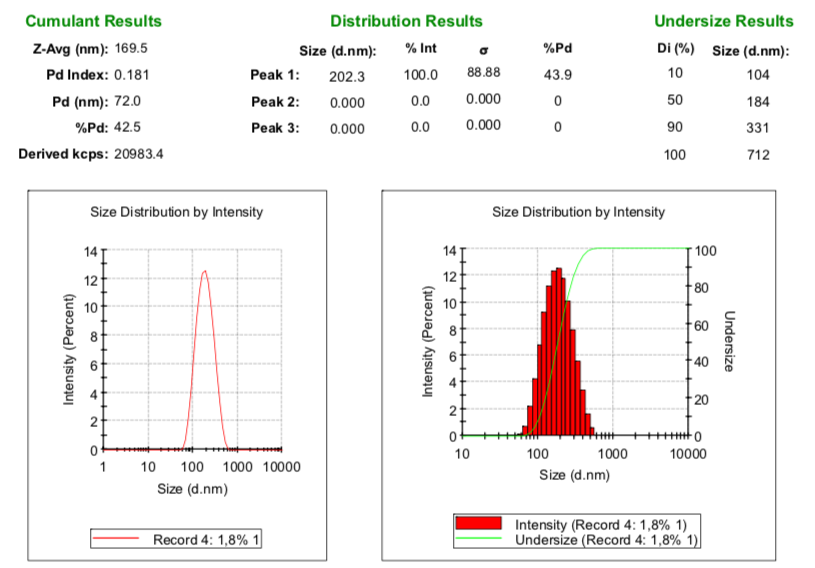
**

**F1 no.2**


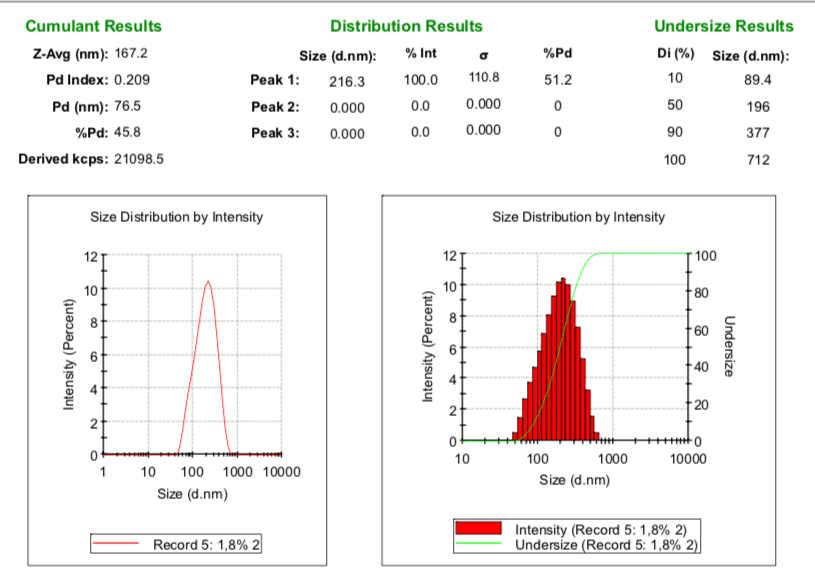


**F1 no. 3**


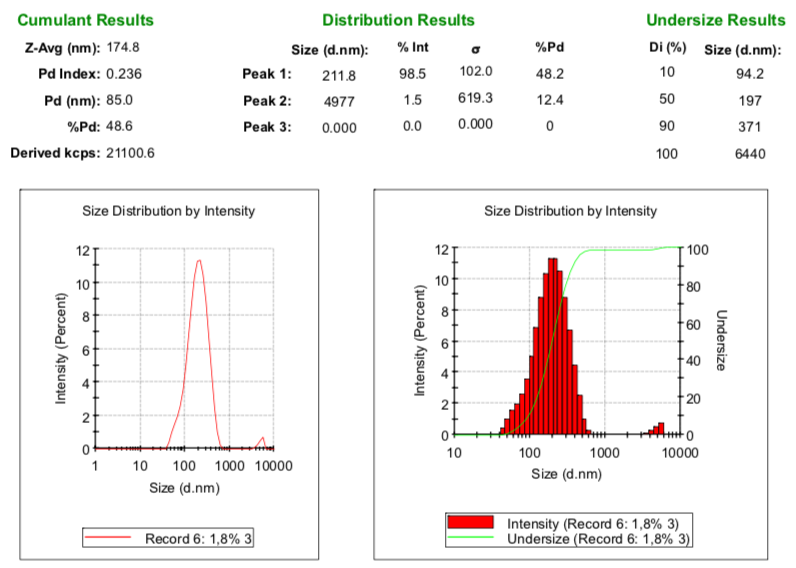


**F2 no 1**


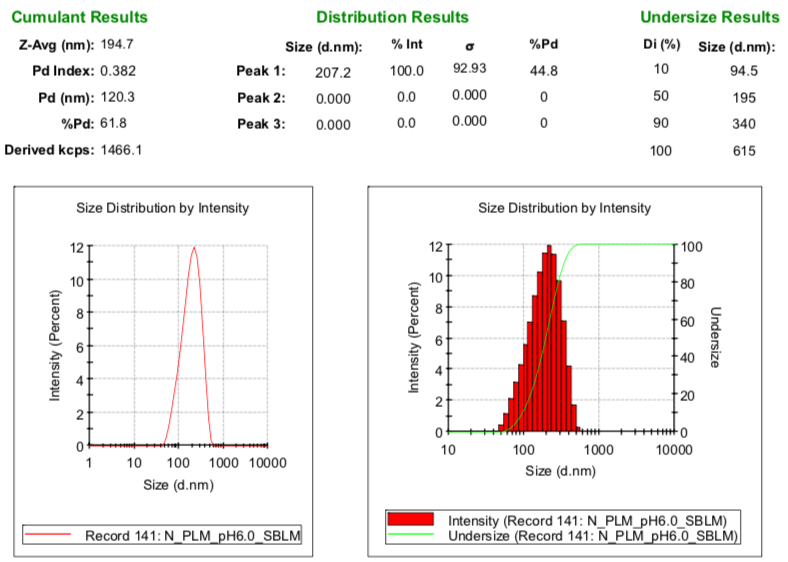


**F2 no 2**


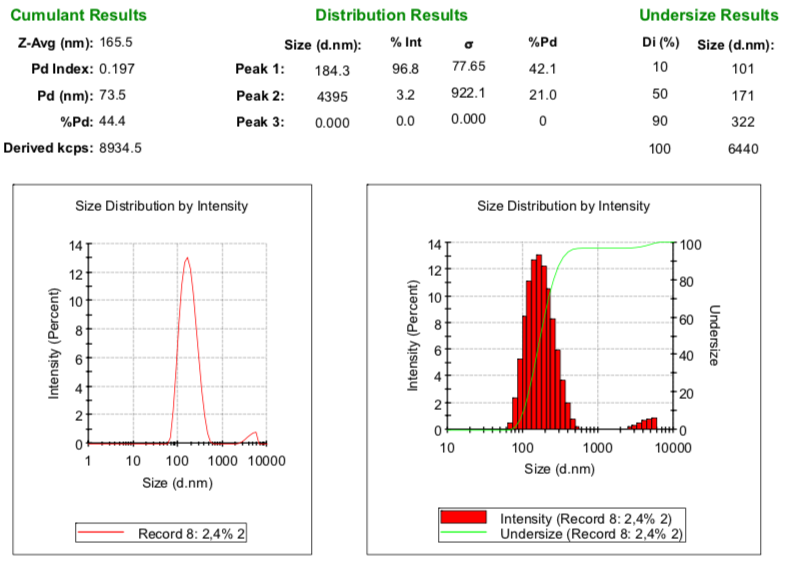


**F2 no 3**


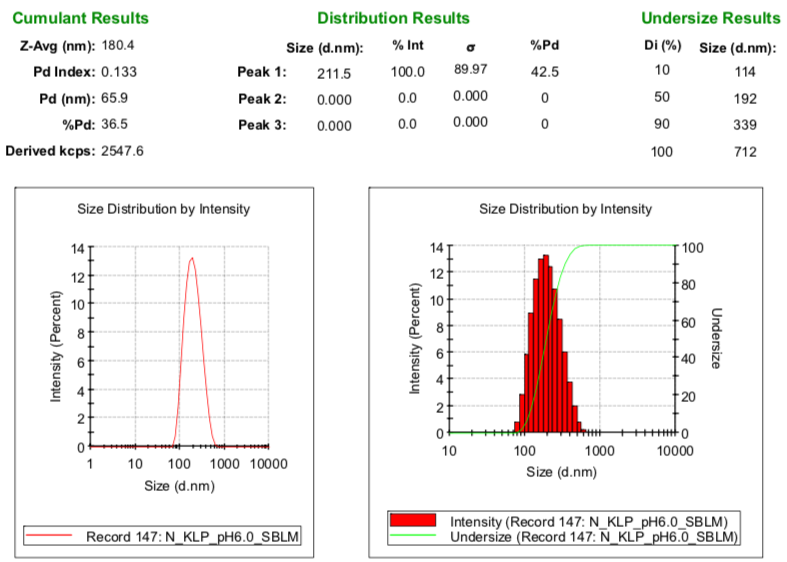


**F3 no 1**


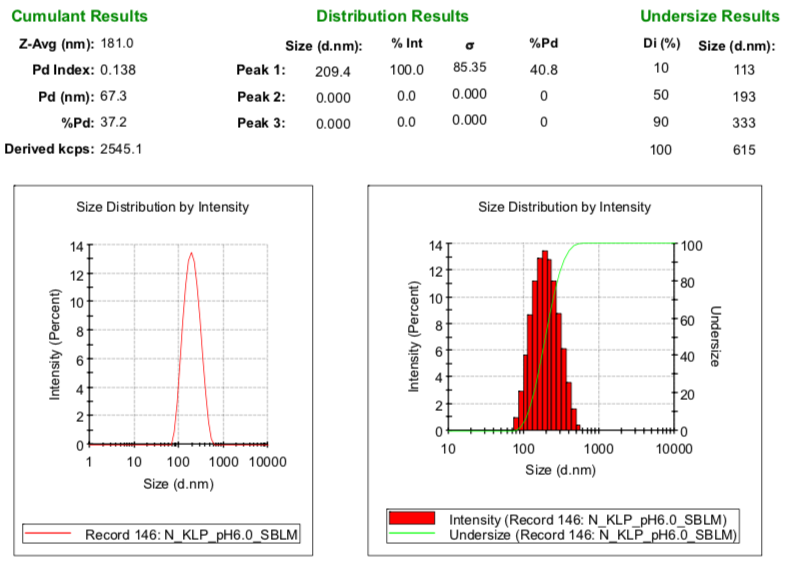


**F3 no 2**


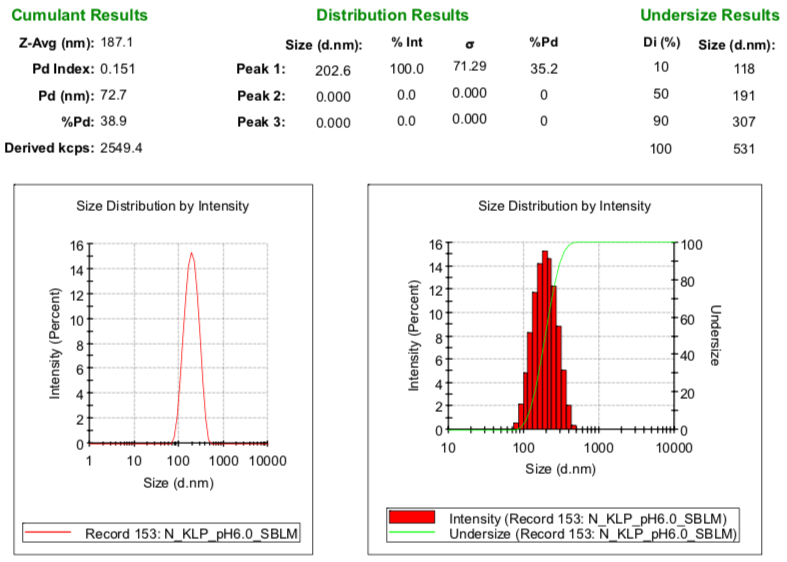


**F3 no 3**


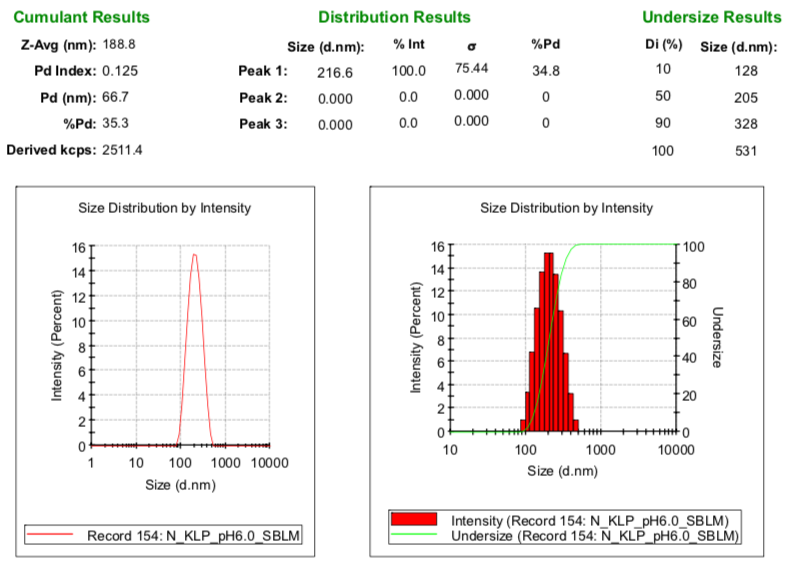


**F4 no 1**


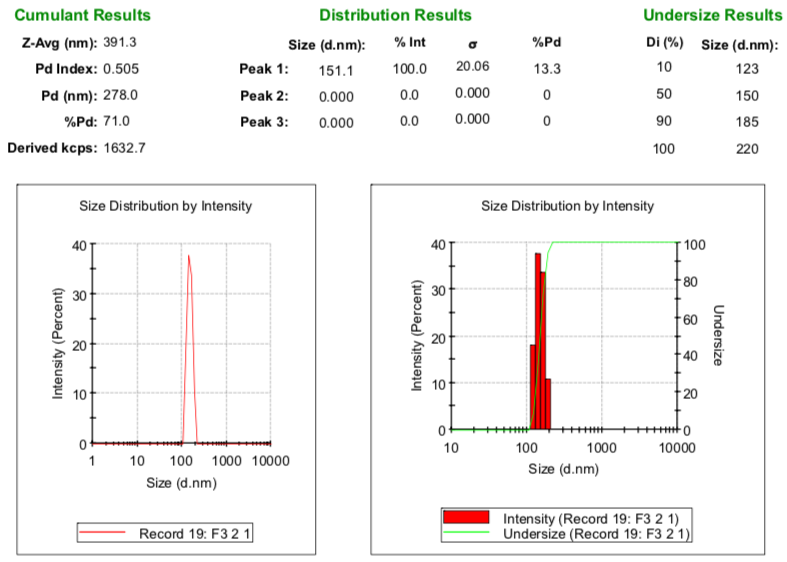


**F4 no 2**


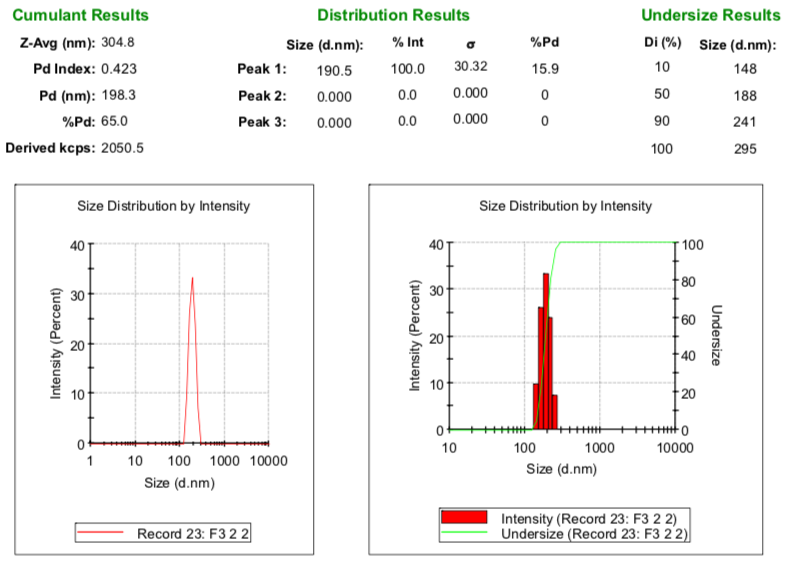


**F4 no 3**


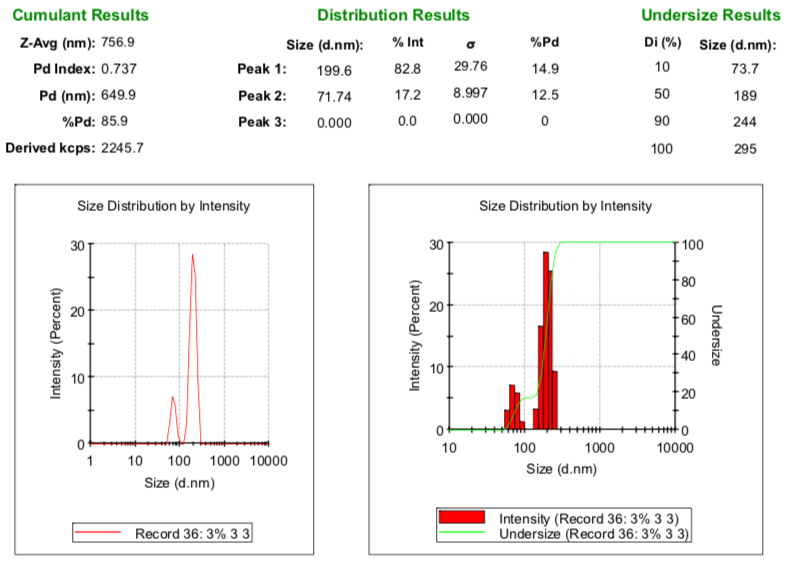


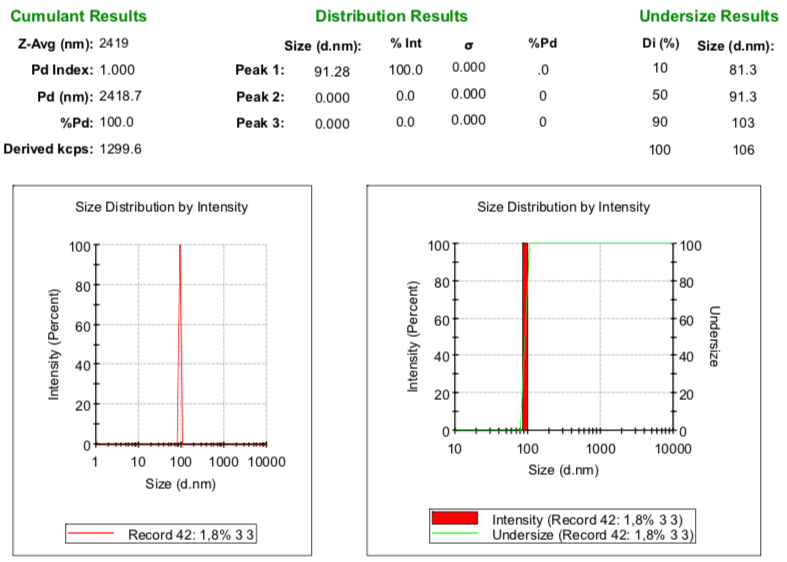


**F5 no 1**


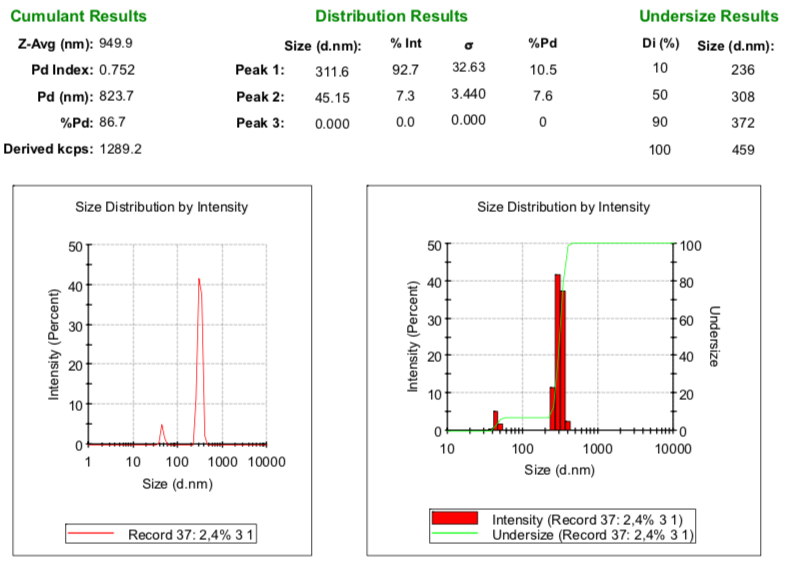


**F5 no 2**


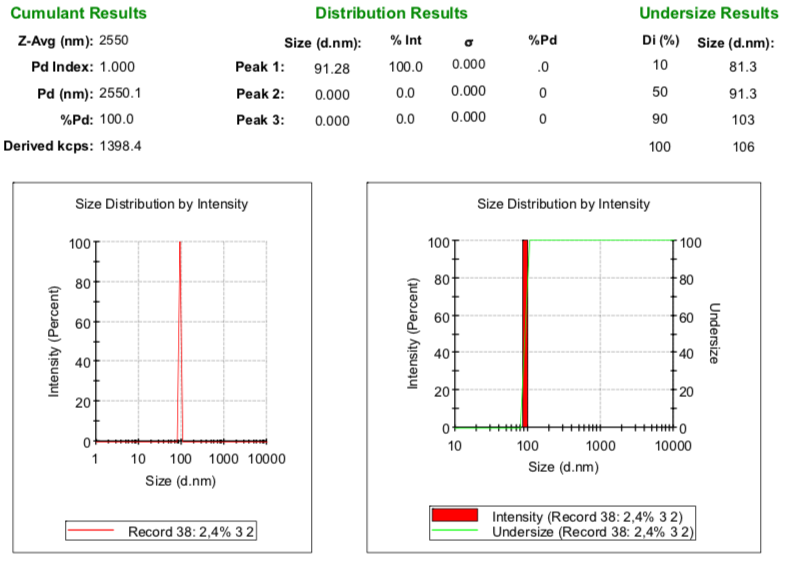


**F5 no 3**


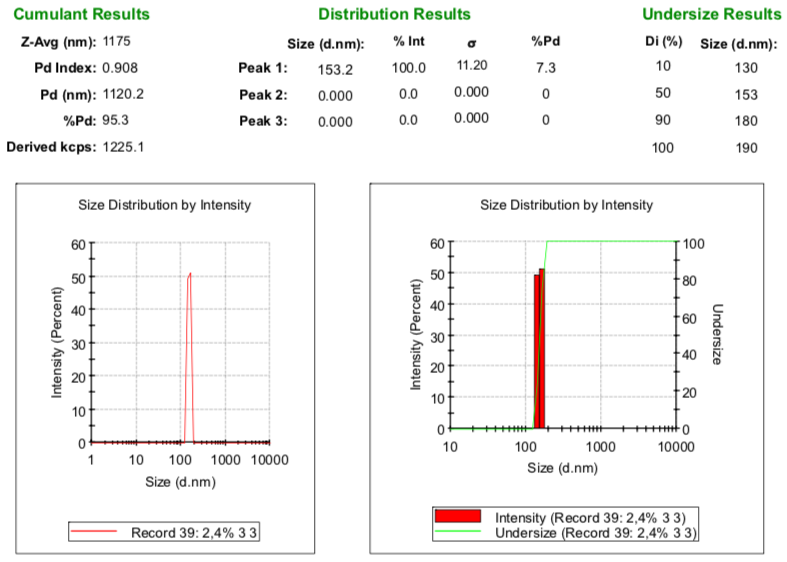


**F6 no 1**


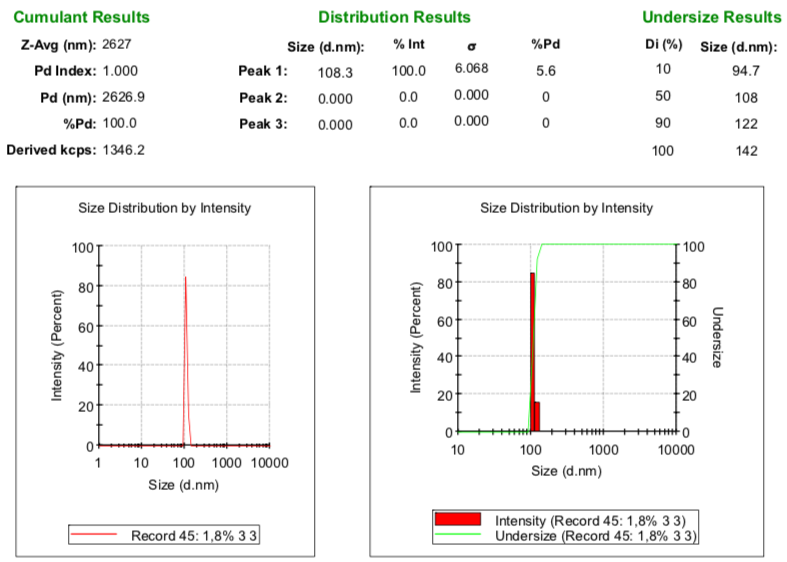


**F6 no 2**


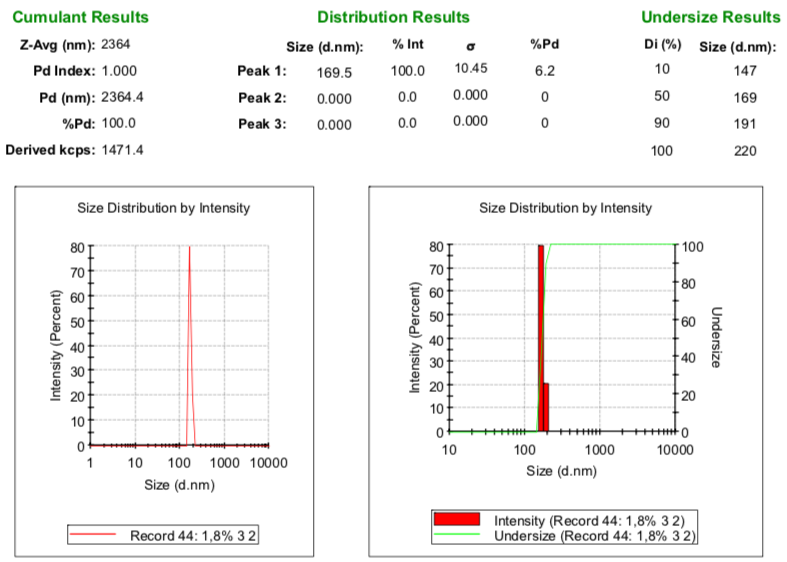


**F6 no 3**


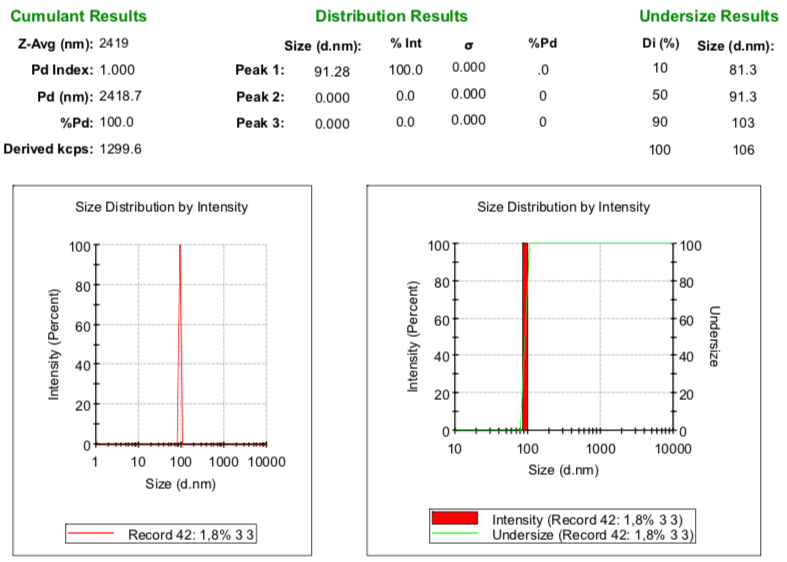


**Appendix 3. Zeta Potential**

| Sample | Zeta Potential | Average | SD |
| --- | --- | --- | --- |
| F1 | -26.3 | -28.43 | 2.38 |
|  | -28 |  |  |
|  | -31 |  |  |
| F2 | -34.5 | -35.27 | 0.68 |
|  | -35.8 |  |  |
|  | -35.5 |  |  |
| F3 | -40.9 | -41.20 | 0.98 |
|  | -40.4 |  |  |
|  | -42.3 |  |  |
| F4 | -29.6 | -28.03 | 2.80 |
|  | -29.7 |  |  |
|  | -24.8 |  |  |
| F5 | -24.8 | -27.33 | 2.27 |
|  | -29.2 |  |  |
|  | -28 |  |  |
| F6 | -20.6 | -23.57 | 2.74 |
|  | -24.1 |  |  |
|  | -26 |  |  |

**Appendix 4. Particle size, Polidispersity Index (PDI), and pH Data Before and After Sterilization of Formula F3.**

| **Parameter** | **n** | **Before** | **After** | **Statistical Analysis** |
| --- | --- | --- | --- | --- |
| Particle Size | 1 | 275 | 333 | 0.055 |
|  | 2 | 278 | 307 |  |
|  | 3 | 281 | 328 |  |
|  | Mean | 278 + 3 | 322.67 + 11.26 |  |
| PDI | 1 | 0.093 | 0.130 | 0.094 |
|  | 2 | 0.083 | 0.151 |  |
|  | 3 | 0.152 | 0.125 |  |
|  | Mean | 0.11 + 0.04 | 0.13 + 0.01 |  |
| pH | 1 | 6.25 | 6.04 | 0.154 |
|  | 2 | 6.28 | 6.10 |  |
|  | 3 | 6.27 | 6.01 |  |
|  | Mean | 6.27 + 0.02 | 6.05 + 0.05 |  |
| Statistical test using Independent Sample Test *p<0.05 | | | | |

**Appendix 5. Viscosity**

1. **Data Viscosity Formula F1, F2, F3, F4, F5, and F6.**

| **Sample** | **Viscosity Replication** | | | **Mean** | **STD** |
| --- | --- | --- | --- | --- | --- |
|  | **1** | **2** | **3** |  |  |
| F1 | 5.8 | 6.14 | 5.97 | 5.97 | 0.17 |
| F2 | 6.43 | 6.64 | 6.22 | 6.43 | 0.21 |
| F3 | 6.38 | 6.88 | 6.63 | 6.63 | 0.25 |
| F4 | 15.53 | 15.32 | 15.11 | 15.32 | 0.21 |
| F5 | 16.5 | 16.27 | 16.04 | 16.27 | 0.23 |
| F6 | 18.89 | 18.54 | 19.24 | 18.89 | 0.35 |

**Apppendix 6. Particle Size, PDI, Zeta Potential, Viscosity, pH, and Curcumin Content Stability Test Data**

1. **Characterization of formula 3**

| Time | n | Particle Siza | PDI | Zeta | Viscosity | pH | Curcumin Content |
| --- | --- | --- | --- | --- | --- | --- | --- |
| 0 Month | 1 | 333 | 0.138 | -40.9 | 6.88 | 6.04 | 99.33% |
|  | 2 | 307 | 0.151 | -40.4 | 6.63 | 6.10 | 102.42% |
|  | 3 | 328 | 0.125 | -42.3 | 6.38 | 6.01 | 97.15% |
|  | Mean | 322.67 | 0.13 | -41.2 | 6.63 | 6.05 | 99.63% |
|  | STD | 11.26 | 0.01 | 0.98 | 0.25 | 0.05 | 2.65% |
| 1 Month | 1 | 327 | 0.310 | -38.40 | 6.57 | 6.01 | 99.02% |
|  | 2 | 349 | 0.345 | -38.70 | 7.19 | 6.03 | 98.59% |
|  | 3 | 367 | 0.232 | -38.9 | 6.88 | 5.97 | 99.81% |
|  | Mean | 347.67 | 0.29 | -38.7 | 6.88 | 6.00 | 99.14% |
|  | STD | 20.03 | 0.05 | 0.25 | 0.31 | 0.03 | 0.62% |
| 2 Month | 1 | 335 | 0.33 | -37.9 | 7.56 | 5.84 | 96.86% |
|  | 2 | 347 | 0.326 | -38.1 | 7.83 | 5.92 | 96.34% |
|  | 3 | 364 | 0.326 | -38 | 7.29 | 5.90 | 99.36% |
|  | Mean | 348.67 | 0.33 | -38 | 7.56 | 5.89 | 97.52% |
|  | STD | 14.67 | 0.002 | 0.10 | 0.27 | 0.04 | 1.61% |
| 3 Month | 1 | 388 | 0.335 | -36.5 | 8.43 | 5.52 | 95.07% |
|  | 2 | 372 | 0.367 | -37.1 | 8.68 | 5.54 | 99.51% |
|  | 3 | 405 | 0.383 | -37 | 8.18 | 5.44 | 94.97% |
|  | Mean | 388.33 | 0.36 | -36.87 | 8.43 | 5.50 | 96.52% |
|  | STD | 16.50 | 0.024 | 0.32 | 0.25 | 0.05 | 2.59% |

1. **Statistical Analysis**

|  | Between Group | | Sig |
| --- | --- | --- | --- |
| Particle Size | 0 Month | 1 Month | .275 |
|  |  | 2 Month | .275 |
|  |  | 3 Month | .050 |
| PDI | 0 Month | 1 Month | .050 |
|  |  | 2 Month | .050 |
|  |  | 3 Month | .050 |
| Zeta Potential | 0 Month | 1 Month | .050 |
|  |  | 2 Month | .050 |
|  |  | 3 Month | .050 |
| Vicosity | 0 Month | 1 Month | .376 |
|  |  | 2 Month | .376 |
|  |  | 3 Month | .050 |
| pH | 0 Month | 1 Month | .184 |
|  |  | 2 Month | .050 |
|  |  | 3 Month | .050 |
| Curcumin Content | 0 Month | 1 Month | .827 |
|  |  | 2 Month | .827 |
|  |  | 3 Month | .275 |
| Statistical test using Mann-Whitney U Test *p<0.05 | | | |

**Apppendix 7. Paw Edema Volume**

1. **Volume Data of rats paw at 1 to 6 hours after being induced with 0.2 ml of 1% carrageenan in all test groups.**

| Group | N | Volume of Paw Edema | | | | | | |
| --- | --- | --- | --- | --- | --- | --- | --- | --- |
|  |  | Before Induction | 1 hr | 2 hr | 3 hr | 4 hr | 5 hr | 6 hr |
| Negative Control | 1 | 0.020 | 0.031 | 0.038 | 0.044 | 0.050 | 0.047 | 0.040 |
|  | 2 | 0.024 | 0.035 | 0.041 | 0.045 | 0.052 | 0.048 | 0.042 |
|  | 3 | 0.023 | 0.034 | 0.040 | 0.045 | 0.052 | 0.048 | 0.043 |
|  | 4 | 0.024 | 0.033 | 0.039 | 0.046 | 0.053 | 0.048 | 0.043 |
|  | 5 | 0.022 | 0.035 | 0.040 | 0.045 | 0.051 | 0.049 | 0.045 |
|  | Mean | 0.0225 | 0.0335 | 0.0395 | 0.045 | 0.0515 | 0.048 | 0.0425 |
|  | SD | 0.00166 | 0.00166 | 0.00112 | 0.00071 | 0.00112 | 0.00071 | 0.00180 |
| Positive Control | 1 | 0.023 | 0.030 | 0.032 | 0.036 | 0.034 | 0.030 | 0.031 |
|  | 2 | 0.024 | 0.031 | 0.033 | 0.034 | 0.045 | 0.033 | 0.035 |
|  | 3 | 0.023 | 0.029 | 0.031 | 0.031 | 0.035 | 0.031 | 0.029 |
|  | 4 | 0.025 | 0.032 | 0.030 | 0.031 | 0.041 | 0.036 | 0.033 |
|  | 5 | 0.025 | 0.033 | 0.034 | 0.034 | 0.040 | 0.032 | 0.029 |
|  | Mean | 0.024 | 0.031 | 0.032 | 0.0332 | 0.039 | 0.0324 | 0.0314 |
|  | SD | 0.00100 | 0.00158 | 0.00158 | 0.00217 | 0.00453 | 0.00230 | 0.00261 |
| Group I | 1 | 0.022 | 0.029 | 0.034 | 0.040 | 0.043 | 0.041 | 0.038 |
|  | 2 | 0.021 | 0.027 | 0.035 | 0.039 | 0.042 | 0.041 | 0.036 |
|  | 3 | 0.021 | 0.028 | 0.035 | 0.040 | 0.043 | 0.041 | 0.037 |
|  | 4 | 0.022 | 0.031 | 0.034 | 0.037 | 0.040 | 0.035 | 0.037 |
|  | 5 | 0.021 | 0.028 | 0.031 | 0.036 | 0.039 | 0.034 | 0.035 |
|  | Mean | 0.0214 | 0.0286 | 0.0338 | 0.0384 | 0.0414 | 0.0384 | 0.0366 |
|  | SD | 0.00055 | 0.00152 | 0.00164 | 0.00182 | 0.00182 | 0.00358 | 0.00114 |
| Group II | 1 | 0.019 | 0.025 | 0.028 | 0.033 | 0.035 | 0.029 | 0.028 |
|  | 2 | 0.022 | 0.027 | 0.031 | 0.035 | 0.039 | 0.032 | 0.033 |
|  | 3 | 0.023 | 0.028 | 0.032 | 0.035 | 0.038 | 0.037 | 0.038 |
|  | 4 | 0.021 | 0.029 | 0.033 | 0.037 | 0.040 | 0.031 | 0.031 |
|  | 5 | 0.020 | 0.025 | 0.029 | 0.033 | 0.036 | 0.032 | 0.032 |
|  | Mean | 0.021 | 0.0268 | 0.0306 | 0.0346 | 0.0376 | 0.032 | 0.0324 |
|  | SD | 0.00158 | 0.00179 | 0.00207 | 0.00167 | 0.00207 | 0.00295 | 0.00365 |

1. **Statistical nalysis results on the volume data of rats paw at 1 to 6 hours after being induced with 0.2 ml of 1% carrageenan in all test groups.**

| Between Group | | Time | | | | | | |
| --- | --- | --- | --- | --- | --- | --- | --- | --- |
|  |  | Before Induction | ^(a)^1 hr | ^(a)^2 hr | ^(a)^3 hr | ^(b)^4 hr | ^(b)^5 hr | ^(a)^6 hr |
| Negative Control | Positive Control | ^a^0.104 | 0.024* | 0.000* | 0.000* | 0.009* | 0.008* | 0.000* |
|  | Group I | ^b^0.163 | 0.000* | 0.000* | 0.000* | 0.009* | 0.007* | 0.002* |
|  | Group II | ^a^0.066 | 0.000* | 0.000* | 0.000* | 0.009* | 0.008* | 0.000* |
| Positive Control | Group I | ^b^0.008* | 0.035* | 0.102 | 0.000* | 0.401 | 0.026* | 0.004* |
|  | Group II | ^a^0.002* | 0.001* | 0.197 | 0.206 | 0.599 | 0.832 | 0.534 |
| Group I | Group II | ^a^0.629 | 0.102 | 0.007* | 0.003* | 0.027* | 0.026* | 0.017* |
| ^a^ Statistical test using LSD Tukey Test  ^b^ Statistical test using Mann-Whitney U Test  *p<0.05 | | | | | | | | |

**Apppendix 8. Paw Edema Inhibition Inflammation**

1. **Percent (%) of inhibition data of the rats paw edema at the 1 to 6 hours after being induced by 0.2 ml of 1% carrageenan in all test groups.**

| Group | N | % Inhibition Paw Edema | | | | | |
| --- | --- | --- | --- | --- | --- | --- | --- |
|  |  | 1 hr | 2 hr | 3 hr | 4 hr | 5 hr | 6 hr |
| Positive Control | 1 | 36.36% | 47.06% | 42.22% | 62.07% | 72.55% | 60.00% |
|  | 2 | 36.36% | 47.06% | 55.56% | 27.59% | 64.71% | 45.00% |
|  | 3 | 45.45% | 52.94% | 64.44% | 58.62% | 68.63% | 70.00% |
|  | 4 | 36.36% | 70.59% | 73.33% | 44.83% | 56.86% | 60.00% |
|  | 5 | 27.27% | 47.06% | 60.00% | 48.28% | 72.55% | 80.00% |
|  | Mean | 36.36% | 52.94% | 59.11% | 48.28% | 67.06% | 63.00% |
|  | SD | 6.43% | 10.19% | 11.50% | 13.58% | 6.56% | 13.04% |
| Group I | 1 | 36.36% | 29.41% | 20.00% | 27.59% | 25.49% | 20.00% |
|  | 2 | 45.45% | 17.65% | 20.00% | 27.59% | 21.57% | 25.00% |
|  | 3 | 36.36% | 17.65% | 15.56% | 24.14% | 21.57% | 20.00% |
|  | 4 | 18.18% | 29.41% | 33.33% | 37.93% | 49.02% | 25.00% |
|  | 5 | 36.36% | 41.18% | 33.33% | 37.93% | 49.02% | 30.00% |
|  | Mean | 34.55% | 27.06% | 24.44% | 31.03% | 33.33% | 24.00% |
|  | SD | 9.96% | 9.84% | 8.31% | 6.45% | 14.41% | 4.18% |
| Group II | 1 | 45.45% | 47.06% | 37.78% | 44.83% | 60.78% | 55.00% |
|  | 2 | 54.55% | 47.06% | 42.22% | 41.38% | 60.78% | 45.00% |
|  | 3 | 54.55% | 47.06% | 46.67% | 48.28% | 45.10% | 25.00% |
|  | 4 | 27.27% | 29.41% | 28.89% | 34.48% | 60.78% | 50.00% |
|  | 5 | 54.55% | 47.06% | 42.22% | 44.83% | 52.94% | 40.00% |
|  | Mean | 47.27% | 43.53% | 39.56% | 42.76% | 56.08% | 43.00% |
|  | SD | 11.85% | 7.89% | 6.74% | 5.23% | 7.02% | 11.51% |

1. **Statistical analysis result on the Percent (%) of inhibition data of the rats paw edema at the 1 to 6 hours after being induced by 0.2 ml of 1% carrageenan in all test groups.**

| Between Group | | Time | | | | | |
| --- | --- | --- | --- | --- | --- | --- | --- |
|  |  | 1 hr | ^(b)^2 hr | ^(b)^3 hr | ^(b)^4 hr | ^(b)^5 hr | ^(b)^6 hr |
| Positive Control | Group I | ^a^0.798 | 0.008* | 0.093 | 0.209 | 0.089 | 0.036* |
|  | Group II | ^b^0.026* | 0.095 | 0.106 | 0.289 | 0.209 | 0.044* |
| Group I | Group II | ^b^0.015* | 0.156 | 0.527 | 0.173 | 0.025 | 0.016* |
| ^a^ Statistical test using LSD Tukey Test  ^b^ Statistical test using Mann-Whitney U Test  *=P<0.05 | | | | | | | |

**Apppendix 9. Paw Edema Diameter**

1. **Diameter Data of the rats paw edema at the 1 to 6 hours after being induced by 0.2 ml of 1% carrageenan in all test groups.**

| Group | N | **Diameter of Paw Edema** | | | | | | |
| --- | --- | --- | --- | --- | --- | --- | --- | --- |
|  |  | Before Induction | 1 hr | 2 hr | 3 hr | 4 hr | 5 hr | 6 hr |
| Negative Control | 1 | 3.30 | 4.50 | 5.80 | 6.40 | 6.80 | 6.10 | 7.40 |
|  | 2 | 3.50 | 5.30 | 5.60 | 6.60 | 7.50 | 6.60 | 7.80 |
|  | 3 | 3.40 | 5.00 | 5.60 | 7.20 | 7.50 | 6.00 | 8.00 |
|  | 4 | 3.30 | 5.10 | 6.40 | 6.90 | 7.80 | 7.70 | 8.00 |
|  | 5 | 3.40 | 5.60 | 6.10 | 7.00 | 7.40 | 6.80 | 7.90 |
|  | Mean | 3.380 | 5.100 | 5.900 | 6.820 | 7.400 | 6.640 | 7.820 |
|  | SD | 0.084 | 0.406 | 0.346 | 0.319 | 0.367 | 0.680 | 0.249 |
| Positive Control | 1 | 3.50 | 3.70 | 4.20 | 4.60 | 4.70 | 4.40 | 4.60 |
|  | 2 | 3.40 | 3.50 | 4.80 | 5.50 | 5.90 | 5.40 | 5.40 |
|  | 3 | 3.30 | 3.50 | 4.30 | 5.20 | 5.50 | 5.50 | 5.00 |
|  | 4 | 3.00 | 3.30 | 4.40 | 5.20 | 5.80 | 5.40 | 5.00 |
|  | 5 | 2.90 | 3.20 | 3.40 | 4.50 | 4.90 | 4.70 | 4.00 |
|  | Mean | 3.220 | 3.440 | 4.220 | 5.000 | 5.360 | 5.080 | 4.800 |
|  | SD | 0.259 | 0.195 | 0.512 | 0.430 | 0.537 | 0.497 | 0.529 |
| Group I | 1 | 3.00 | 3.60 | 5.20 | 5.20 | 4.80 | 4.80 | 5.00 |
|  | 2 | 3.00 | 3.90 | 4.40 | 5.20 | 5.50 | 5.50 | 5.70 |
|  | 3 | 3.30 | 3.80 | 5.10 | 5.90 | 5.70 | 5.30 | 5.60 |
|  | 4 | 3.00 | 3.80 | 3.80 | 5.60 | 5.20 | 5.00 | 5.60 |
|  | 5 | 3.40 | 3.90 | 5.50 | 5.40 | 5.70 | 6.60 | 6.80 |
|  | Mean | 3.140 | 3.800 | 4.800 | 5.460 | 5.380 | 5.440 | 5.740 |
|  | SD | 0.195 | 0.122 | 0.689 | 0.297 | 0.383 | 0.702 | 0.654 |
| Group II | 1 | 3.40 | 3.90 | 5.30 | 5.70 | 6.60 | 5.70 | 5.20 |
|  | 2 | 3.40 | 3.80 | 3.50 | 4.20 | 5.30 | 4.90 | 4.50 |
|  | 3 | 3.10 | 3.60 | 4.20 | 4.50 | 4.50 | 4.90 | 4.60 |
|  | 4 | 3.30 | 3.90 | 4.20 | 4.30 | 6.20 | 5.80 | 5.20 |
|  | 5 | 3.10 | 3.70 | 4.30 | 4.30 | 5.50 | 5.20 | 4.30 |
|  | Mean | 3.260 | 3.780 | 4.300 | 4.600 | 5.620 | 5.300 | 4.760 |
|  | SD | 0.152 | 0.130 | 0.644 | 0.624 | 0.817 | 0.430 | 0.416 |

1. **Statistical analysis result on the diameter data of the rats paw edema at the 1 to 6 hours after being induced by 0.2 ml of 1% carrageenan in all test groups.**

| Between Group | | Time | | | | | | |
| --- | --- | --- | --- | --- | --- | --- | --- | --- |
|  |  | ^(b)^Before Induction | ^(a)^1 hr | ^(a)^2 hr | 3 hr | ^(a)^4 hr | ^(a)^5 hr | ^(a)^6 hr |
| Negative Control | Positive Control | 0.390 | 0.000* | 0.000* | ^a^0.000* | 0.000* | 0.001* | 0.000* |
|  | Group I | 0.065 | 0.000* | 0.007* | ^b^0.009* | 0.000* | 0.005* | 0.000* |
|  | Group II | 0.228 | 0.000* | 0.000* | ^b^0.009* | 0.000* | 0.002* | 0.000* |
| Positive Control | Group I | 0.664 | 0.032* | 0.123 | ^a^0.116 | 0.955 | 0.348 | 0.007* |
|  | Group II | 0.831 | 0.041* | 0.825 | ^b^0.140 | 0.470 | 0.563 | 0.898 |
| Group I | Group II | 0.196 | 0.898 | 0.180 | ^b^0.074 | 0.505 | 0.712 | 0.006* |
| ^a^ Statistical test using LSD Tukey Test  ^b^ Statistical test using Mann-Whitney U Test  *=P<0.05*=P<0.05 | | | | | | | | |
